# A Space Efficient Flexible Pivot Selection Approach to Evaluate Determinant and Inverse of a Matrix

**DOI:** 10.1371/journal.pone.0087219

**Published:** 2014-02-03

**Authors:** Hafsa Athar Jafree, Muhammad Imtiaz, Syed Inayatullah, Fozia Hanif Khan, Tajuddin Nizami

**Affiliations:** 1 Department of Mathematical Sciences, University of Karachi, Karachi, Pakistan; 2 Department of Mathematics, Sir Syed University of Engineering and Technology, Karachi, Pakistan; 3 Department of Mathematics, Iqra University, Karachi, Pakistan; University of Nottingham, United Kingdom

## Abstract

This paper presents new simple approaches for evaluating determinant and inverse of a matrix. The choice of pivot selection has been kept arbitrary thus they reduce the error while solving an ill conditioned system. Computation of determinant of a matrix has been made more efficient by saving unnecessary data storage and also by reducing the order of the matrix at each iteration, while dictionary notation [1] has been incorporated for computing the matrix inverse thereby saving unnecessary calculations. These algorithms are highly class room oriented, easy to use and implemented by students. By taking the advantage of flexibility in pivot selection, one may easily avoid development of the fractions by most. Unlike the matrix inversion method [2] and [3], the presented algorithms obviate the use of permutations and inverse permutations.

## Introduction

The term determinant is originally associated with the system of linear equations. It provides us with a foresight about the nature of solution of a given system of linear equations.

Maclaurin [4] published the first result on determinant of 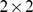 and 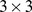 system, which was generalized for 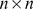 systems by Cramer [5]. Later, the well known Laplace expansion for evaluating determinant was proposed by Laplace, but he had then used the term ‘resultant’ instead of determinant. The first use of the term determinant in the modern context was done by Cauchy [6]. In 1866, Dodgson presented another method for finding the determinant of 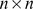 systems which he named as the “Method of condensation” [7]. For larger systems the most preferred method for evaluating the determinant up till now is the Gaussian method [8]. It deals with the problem by converting the coefficient matrix into its equivalent upper/lower triangular form. The product of the pivot elements gives the determinant.

The existence of matrix inverse also depends on its determinant. The method of finding the inverse by Gaussian method is discussed later in this paper. Ahmed & Khan [2], and Khan, Shah, & Ahmad [3] proposed algorithms for the calculation of inverse of a matrix which are streamlined forms of the Gaussian method and also require permutation and inverse permutations. In this paper, we have presented two new algorithms related to the evaluation of determinant and inverse of a matrix. The first presented algorithm evaluates the determinant and is more efficient than Gaussian method as it reduces the order of the matrix at each iteration thereby saving unnecessary computations. In the second algorithm, we have presented another easy to mange way to calculate the inverse of the matrix by constructing the dictionary of the given system and thus excluding the need of permutations and inverse permutations.

## Determinant of A Matrix: A Brief Review

Determinant of a square matrix **A**, denoted by *det*(**A**), is basically a real valued function. Because of its useful relationship with the matrix **A** and the solution to the system of equations of the form **A** *x* = *b*, it becomes essential to have a knowledge about determinants while studying matrices. Evaluation of a determinant through its cofactor expansion (also known as Laplace expansion) is famous for lower order 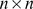 matrices.
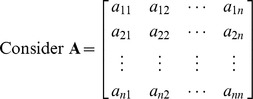



Let 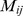 be the minor of entry 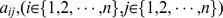 which is the determinant of the sub-matrix obtained after deleting the 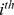 row and 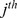 column of **A**.

If 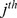 column of **A** is opted for cofactor expansion then,
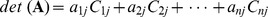
where, 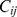 is the cofactor of entry 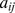 such that 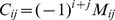.

Similarly the cofactor expansion along the 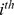 row would be
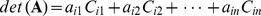



For a matrix of order *n*, the evaluation of determinant by the above cofactor expansion requires computing *n* determinants of matrices of order (*n*−1). Therefore it can be implemented for finding determinant of a matrix of order 2 or 3 with ease but for higher orders it becomes a tedious job. To reduce the computational effort usually the following three basic row operations of matrices are incorporated to evaluate the determinant [9], the method is known as evaluation of determinant by row reduction (also known as Gaussian method).

### Elementary row operations

Let **A** be an 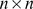 matrix, the following elementary row operations can be applied

- Multiply a row by a non-zero constant.
- Interchange two rows.
- Add a multiple of one row to another row.

### Effect of row operations on the value of determinant: [9]

Let **A** be an 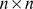 matrix then

- If **B** is the matrix that results when a single row or single column of **A** is multiplied by a scalar k, then det(**B**) = k det(**A**).
- If **B** is the matrix that results when a two rows or two columns of **A** are interchanged then det(**B**) = −det(**A**).
- If **B** is the matrix that results when a multiple of one row of **A** is added to another row or when a multiple of one column is added to another column, then det(**B**) = det(**A**).

### Lemma: [9]

If **A** is an 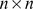 triangular matrix (upper triangular, lower triangular or diagonal), then det(**A**) is the product of the entries on the main diagonal of the matrix; that is, 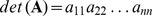.

### Evaluation of determinant by row reduction

The essence of the method is to transform the given matrix into its upper/lower triangular form by applying elementary row operations. The determinant can then be computed by incorporating the properties defined above in ‘Effect of row operations’ and the lemma.

#### Example



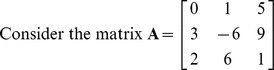


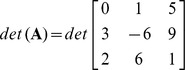


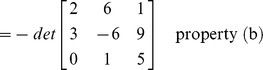


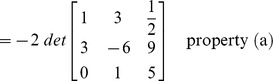


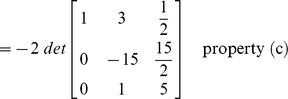


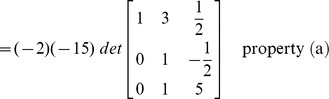


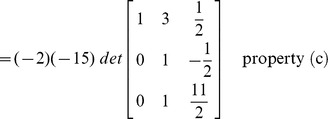


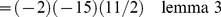


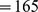



## The New Method

Approach of row reduction may involve all the elementary row operations, as illustrated in above example. Here we are defining an operation (say pivot operation for evaluation of determinant) which consists of (*n*−1) applications of row operation (c) only, and can be employed to evaluate the determinants avoiding involvement of row operations (a) and (b).

For example consider the following matrix,
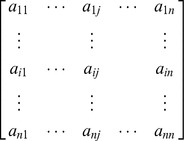



Selecting a non-zero pivot element located at any arbitrary location (*i, j*), say 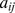, and performing row operation (c) to make the remaining elements of the 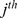 column zero we get
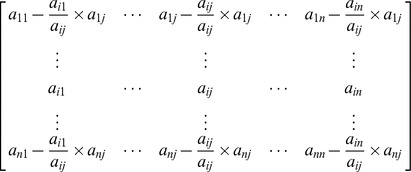


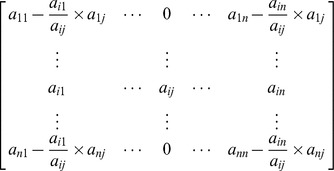



Here we can see that the pivot row remains unchanged while the 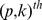 element of the matrix, 
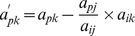
, where 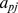 and 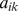 are the elements corresponding to the pivot element in the pivot column and pivot row respectively.

Cofactor expansion along the 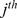 column will give
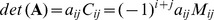


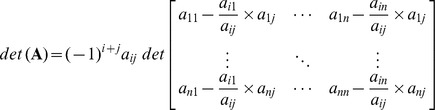



The above procedure results in a computation of determinant of a matrix with reduced order of (*n*−1). A repetition of the above procedure will eventually give a 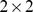 determinant. For a 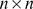 matrix there must be *n* non-zero pivot elements. The product of respective pivot elements gives the value of the determinant. However, if at any step there is no non-zero pivot exists, we can deduce that the determinant of the given matrix is 0.

### Problem 1

Find determinant of the matrix **A** = 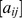 of order 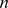.

### Algorithm 1

**Step 1:** Set *d*: = 1,

**Step 2:** set *P*: = {1,2,3,……,*n*}

**Step 3:** Select any *p* 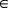 *P* such that *L*: = {*k*: 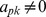, *k* 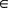 *P*}.

**Step 4:** If *L* = 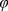 then *d* = 0 and go to step 7

Otherwise 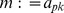 *p* 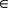 *P*, *k* 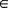 *L*, and
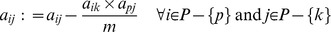



**Step 5:** Reduce the order of **A** by removing 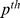 row and 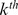 column of **A**. Also set *n*: = *n*−1

**Step 6:** 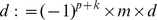. If 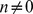 go to step 2.

**Step 7:** *det*(**A**) = *d*. Exit.

### Example



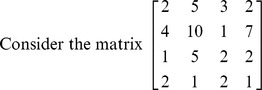



**Iteration 1:**

*P*: = {1,2,3,4}, here we take *p* = 1 so, *L*: = {1,2,3,4}. Taking *k* = 1
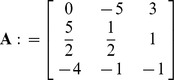


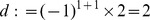



**Iteration 2:**

*P*: = {1,2,3}, here we take *p* = 1 so, *L*: = {2,3}. Taking *k* = 2
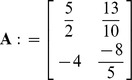


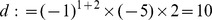



**Iteration 3:**

*P*: = {1,2}, here we take *p* = 1 so, *L*: = {1,2}. Taking *k* = 1
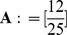


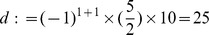



**Iteration 4:**

*P*: = {1}, here we take *p* = 1 so, *L*: = {1}. Taking *k* = 1
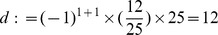
Hence determinant of given matrix is 12.

### Comparison with row reduction method

The above example illustrates the efficiency of the algorithm in terms of memory storage and the number of elements computed. If a matrix of order 4 is solved by row reduction method the number of elements computed will be 12, 6 and 2 in first, second and third iterations respectively. However we have computed 9, 4 and 1 elements in the respective iterations. So the total number of element computations needed for row reduction method is 20, but our method needs only 14 element computations. Also, at each iteration the size of the matrix has been reduced. Row reduction method needs to store 16 elements for each iteration, hence on the whole method requires 48 elements to be stored in the memory, while in contrast our algorithm stores 16+9+4+1 = 30 elements, which is a noteworthy reduction in terms of storage requirement.

A comparison of the number of elements computed and stored to evaluate the determinant of different orders by row reduction and our algorithm are shown in table 1.

**Table 1:** Comparison of row reduction method with algorithm 1 for evaluating determinant of matrix.

Order	Row reduction method		Algorithm 1	
	No. of Element Computations	Storage Requirements (no. of elements)	No. of Element Computations	Storage Requirements (no. of elements)
2	2	8	1	5
4	20	48	14	30
5	40	125	30	55
7	112	343	94	140
10	330	1000	285	385
12	572	1728	506	650

## Inverse of A Matrix: A Brief Review

Consider a matrix 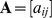 of order *n*. To evaluate the inverse of the matrix, say **B**, one must solve the following *n* system of equations, for 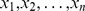


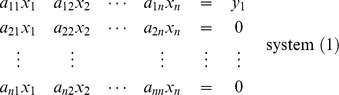


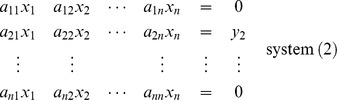


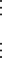


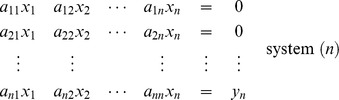
The solutions of the above system are

From system (1) : 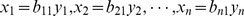


From system (2) : 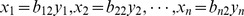


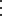
From system (*n*) :
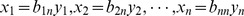


Then the matrix 
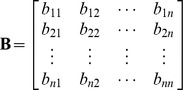
 is called the inverse of matrix **A**.

This procedure could be compactly preformed by expressing the above system in the augmented form,
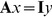
(1)where 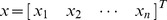 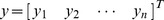 and **I** is identity matrix of order *n*.

If **A** is invertible, applying successive elementary row operations of Gaussian method yields,
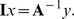



## The New Approach Based on Dictionary Notation

Now consider again the Equation (1) in expanded form,
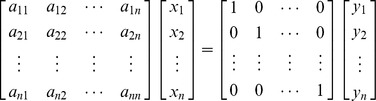
Usually the following augmented matrix is used to solve above system,
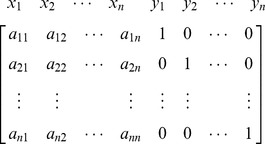
Here, we are using the concept of dictionary notation developed by [1]. Now basic variables are the variables whose coefficients are in the form of any column of identity matrix, and a basis is collection of all basic variables. We can see that basis for the above matrix 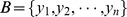 and we may consider 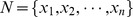 as the non-basis of the current matrix. The objective is to convert the basic variables into non-basic variables and vice versa by using pivot operations. Using the dictionary concept defined by [1] we can remove the basic columns from the matrix and construct the following dictionary form with basis *B* and non-basis *N*: (Note basic variables are shown in left most column and non basic variables in the top row of the dictionary).
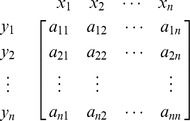



### Pivot operation for evaluating inverse

The following pivot operations [1] may be applied to enter *x_i_* into basis *B* and *y_j_* into non-basis *N*,

- Divide the pivot row by pivot element and the pivot column by negative of the pivot element (except the pivot element).
- The remaining (*n*−1)^2^ elements are determined by the formula as defined in the new method of determinant.
- Reciprocate the pivot element.

If the number of pivot elements is equal to the order of the matrix the resulting matrix gives the inverse otherwise we may conclude that the inverse does not exist.

### Problem 2

Find the inverse of matrix **A** = 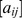 of order 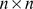.

### Algorithm 2

**Step 1:** Set *H*: = {1,2,3,……,*n*}, *B*: = 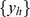 and *N*: = 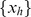, 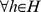. Construct dictionary of the matrix **A**, i.e. *D*(**A**).

**Step 2:** Set *P*: = {*p*: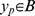}

**Step 3:** If *P* = 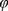, go to step 6.

Otherwise, *L*: = {*k*: 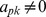, *x_k_* 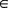 *N*}

**Step 4:** If *L* = 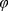 then inverse does not exist. Exit

Otherwise, for any 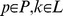


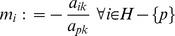


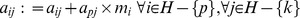


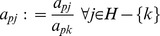


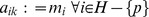


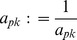



**Step 5:** 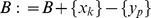 and 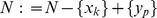. Update *D*(**A**) and go to step 2.

**Step 6:** *Inv*(**A**) = 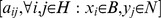 Exit.

### Example



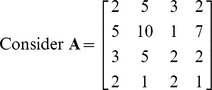


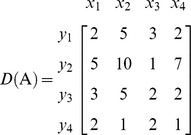
Here *N*: = {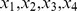}, *B*: = {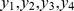}

**Iteration 1:**

*H*: = {1,2,3,4}, *P*: = {1,2,3,4}, taking *p* = 1 we get *L*: = {1,2,3,4}. Taking *k* = 1
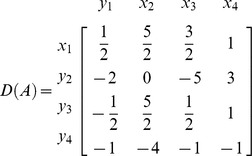

*N*: = {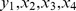}, *B*: = {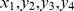}

**Iteration 2:**

*P*: = {2,3,4}, taking *p* = 2 we get *L*: = {3,4}. Taking *k* = 3
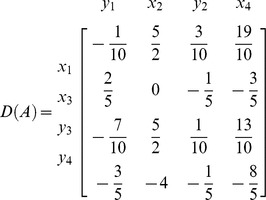

*N*: = {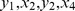}, *B*: = {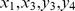}

**Iteration 3:**

*P*: = {3,4}, taking *p* = 3 we get *L*: = {2,4}. Taking *k* = 2
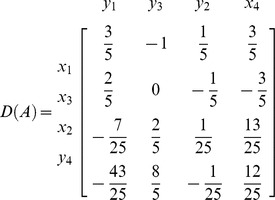

*N*: = {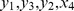}, *B*: = {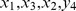}

**Iteration 4:**

*P*: = {4}, taking *p* = 4 we get *L*: = {4}. Taking *k* = 4
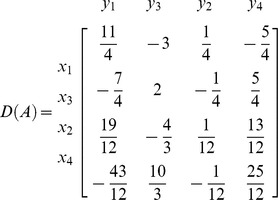

*N*: = {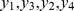}, *B*: = {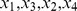}

Now place the elements with respect to indices of variables in *B* and *N*.

For example Here *H*: = {1,2,3,4}, So 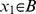 and 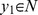, implies 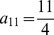. Also 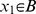 and 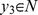 implies 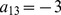. Similarly placing the remaining elements we get
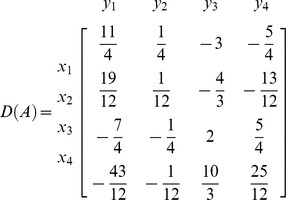



### Comparison with Gaussian method

The above example illustrates the efficiency of the algorithm in terms of memory storage and the number of elements computed. If a matrix of order 4 is solved by Gaussian method the number of elements computed will be 20 at each iteration. However our method requires computation of 16 elements at each iteration. So the total number of element computations needed for Gaussian method is 80, on the other hand for our method it is 64. Also the Gaussian method needs to store 32 elements for each iteration, hence on the whole Gaussian method requires 128 elements to be stored in the memory, while in contrast our algorithm stores 64 elements, which is a noteworthy reduction in terms of storage requirement.

A comparison of the number of elements computed and stored to evaluate the inverse by Gaussian method and our algorithm are shown in table 2.

**Table 2:** Comparison of Gaussian method with the algorithm 2 for evaluating inverse of matrix.

Order	Gaussian Method		Algorithm 2	
	No. of Element Computations	Storage Requirements (no. of elements)	No. of Element Computations	Storage Requirements (no. of elements)
2	12	8	8	4
4	80	32	64	16
5	150	50	125	25
7	392	98	343	49
10	1100	200	1000	100
12	1872	288	1728	144

## Applications

Matrix determinant and inverse have applications in various fields like mathematics, economics, physics, biology etc. Solving different models like population growth involve the use of matrix determinant and inverse. Matrix inverse and determinant are also used in cryptography [10]. Linear transformations (rotation, reflection, translation etc.) involve the calculation of matrix inverse. Matrix inverse and determinant are also employed in operations research while solving linear programs, revised simplex method and markov chains. Determinants of order 3 are used to find area of triangles and for testing colinearity of points. Least square analysis of data requires the evaluation of matrix inverse [11]. *p*-dimensional volume of parallelepiped in 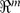 is determined by computing determinant [12].

## Conclusion

This paper presented easy algorithms for computations of determinant and inverse of a matrix. Since the order of the given matrix has been reduced at each step while calculating its determinant, the algorithm reduces the storage requirement (as exhibited in the example). The calculation of inverse has been done using the dictionary notation which obviates the use of permutations and makes it easier to cope with in class room teaching. Ill conditioned system can also be handled as the selection of pivots has been kept arbitrary, thus improving the numerical accuracy of the system.
