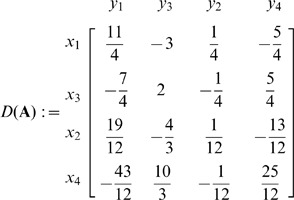# Correction: A Space Efficient Flexible Pivot Selection Approach to Evaluate Determinant and Inverse of a Matrix

**DOI:** 10.1371/journal.pone.0101147

**Published:** 2014-06-18

**Authors:** The PLOS ONE Staff

Several of the equations in this article are incorrect.

In the Determinant of A Matrix: A Brief Review section under the subheading “Evaluation of determinant by row reduction”, the seventh expression is incorrect. The correct expression is:
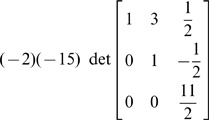



In The New Approach Based on Dictionary Notation section, the example matrices under Step 6 are incorrect. The correct matrices are:
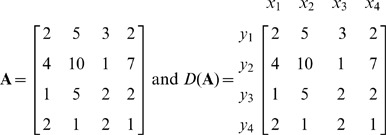



In The New Approach Based on Dictionary Notation section, the dictionary in Iteration 4 is incorrect. The correct dictionary is