# Expression of Concern: A Space Efficient Flexible Pivot Selection Approach to Evaluate Determinant and Inverse of a Matrix

**DOI:** 10.1371/journal.pone.0160281

**Published:** 2016-07-25

**Authors:** The PLOS ONE Editors

After publication of the article readers noted a number of concerns about the algorithm presented in the article. Consultation with editorial board members raised significant concerns relating to the claims of utility, novelty and applicability made about the described method. This re-evaluation revealed that the method is numerically unstable and not a reliable approach to compute determinants in high-quality numerical software. There are no numerical experiments to support the findings and no implementation or comparison of the method to standard tools.

The concerns about the methods and reporting undermine the fulfilment of the journal’s requirements for articles reporting methods. The *PLOS ONE* editors wish to alert readers to the serious limitations of this method that have come to light since publication.
